# Development and Characterization of Innovative Nifurtimox Formulations as Therapeutic Alternative for Chagas Disease

**DOI:** 10.3390/tropicalmed10020050

**Published:** 2025-02-07

**Authors:** Ana Lia Mazzeti, Karolina Ribeiro Gonçalves, Patrícia Ferreira Boasquivis, Maria Terezinha Bahia, Vanessa Carla Furtado Mosqueira

**Affiliations:** 1 Laboratório Integrado de Imunoparasitologia e Farmacotoxicologia, Universidade do Estado de Minas Gerais, Unidade Acadêmica de Passos, Passos 37900-106, MG, Brazil; 2 Laboratório de Doenças Parasitárias, Escola de Medicina & Núcleo de Pesquisas em Ciências Biológicas, Universidade Federal de Ouro Preto, Campus Universitário Morro do Cruzeiro, Ouro Preto 35400-000, MG, Brazil; krgbio@gmail.com (K.R.G.); mtbahia@ufop.edu.br (M.T.B.); 3 Diretoria Industrial, Fundação Ezequiel Dias, Belo Horizonte 30510-010, MG, Brazil; patricia.boasquivis@funed.mg.gov.br; 4 Laboratory of Pharmaceutics and Nanotechnology, School of Pharmacy, Federal University of Ouro Preto, Ouro Preto 35400-000, MG, Brazil; mosqueira@ufop.edu.br

**Keywords:** trypanosomiasis, nifurtimox, self-emulsifying drug delivery systems, poly(ε-caprolactone), implants

## Abstract

Chagas disease, caused by *Trypanosoma cruzi*, remains a neglected tropical disease with limited and often suboptimal chemotherapeutic treatment options. The WHO recommends nifurtimox (NFX) for treating Chagas disease, which, although it is effective in the early stages of infection, has variable efficacy in the chronic phase and induces adverse effects that frequently compromise the continuity of the treatment. This study focused on the development and characterization of innovative lipid-based self-emulsifying drug delivery systems (SEDDSs) and poly(ε-caprolactone) implants containing NFX. The SEDDS formulations modified the NFX release extent and rate. The implant characterization included thermal analysis, X-ray diffraction, thermo-optical analysis, and scanning electron microscopy, confirming the low interaction between NFX and the polymer. In vitro assays demonstrated the enhanced anti-*T. cruzi* activity of the NFX-SEDDS, with minimal cytotoxicity in mammalian cells. In vivo studies using *T. cruzi*-infected mice revealed that both formulations effectively suppressed parasitemia, achieving cure rates comparable to those of the standard oral NFX treatment. Additionally, the implants showed improved tolerability and sustained efficacy, delivering a prolonged effect equivalent to 40 oral doses. These findings highlight the potential of these innovative NFX formulations as promising alternatives for treating Chagas disease, particularly in the chronic phase, offering improved adherence and comparable efficacy to the existing therapies.

## 1. Introduction

Chagas disease, caused by the protozoan *Trypanosoma cruzi* [1,2,3], is a neglected tropical disease affecting approximately 7 million people worldwide [4], with the highest burden in vulnerable regions of Latin America [5]. This life-threatening disease is very complex and involves a stage within the guts of hematophagous insects and other invertebrate hosts, with varied evolutionary extra- and intracellular forms of the protozoan and an ability to infect any type of nucleated human cell. Infective forms of *T. cruzi* are released in the feces and urine of infected Triatomine insects and gain access to the bloodstream of a human host through lesions in the skin or mucous membranes [2,3].

**Figure 1:**
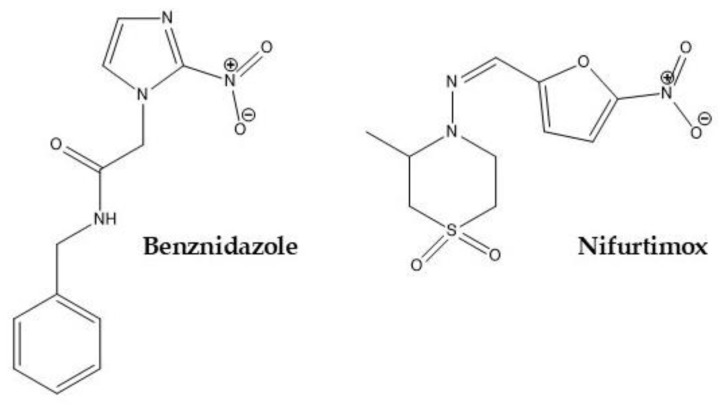
Chemical structures of nitro-heterocyclic compounds, nifurtimox and benznidazole, both recommended by the World Health Organization for treating Chagas disease.

The two drugs recommended by the World Health Organization (WHO) for treating Chagas disease are the nitro-heterocyclic compounds benznidazole (IUPAC name: N-benzyl-2-(2-nitro-1H-imidazol-1-yl)acetamide) (BZN) and nifurtimox (NFX) (IUPAC name: (E)-N-(3-methyl-1,1-dioxo-1,4-thiazinan-4-yl)-1-(5-nitrofuran-2-yl)methanimine) (Figure 1). The precise mechanisms of action of these drugs have not been fully elucidated. Both act as prodrugs activated by *T. cruzi* nitroreductases to induce cytotoxic effects. NFX generates radical species that cause oxidative damage, including thiol depletion, DNA damage, and lipid peroxidation [6,7,8,9]. In contrast, BNZ forms reactive metabolites that alkylate DNA, lipids, and proteins. BNZ may also stimulate immune-mediated parasite killing by increasing the interferon-γ levels due to inflammation. Additionally, BNZ exposure leads to extensive DNA unpacking in parasites and the upregulation of DNA repair proteins, suggesting a DNA-damage-based mechanism [6,7,8,9]. Both are used at high doses for long periods of treatment in Chagas disease. NFX is administered orally at 10–15 mg/kg/day, divided into two or three doses, for 60 days during both the acute and chronic phases of *T. cruzi* infection [10,11].

NFX is also part of the first-line treatment for second-stage gambiense human African trypanosomiasis (HAT), also known as sleeping sickness, administered as part of a combination therapy with eflornithine. HAT is caused by *Trypanosoma brucei* and is considered a serious, neglected parasitic illness [12]. In the combined regimen, NFX is given orally three times daily for 10 days (15 mg/kg per day, every 8 h), while eflornithine is administered intravenously twice daily for 7 days (400 mg/kg per day, every 12 h) [13,14]. This combination therapy requires hospitalization, intensive nursing care, and a complex logistical network for drug transportation.

For Chagas disease, evidence suggests that treatment with BZN or NFX is effective during the early stages of infection. However, its benefit in the late chronic stages remains questionable due to its limited and variable efficacy [11,15,16]. Moreover, the prolonged treatment duration often results in adverse reactions, leading to poor adherence and high dropout rates [17]. Regarding NFX, common adverse effects include gastrointestinal symptoms such as nausea, vomiting, and anorexia, as well as central-nervous-system-related issues like insomnia, irritability, and disorientation. Less frequently reported side effects are headache, rash, myalgia, arthralgia, dizziness, vertigo, and mood changes. Severe but rare adverse effects include polyneuropathy, paresthesia, and peripheral neuritis [17,18]. However, when the treatment with one drug, BZN or NFX, must be discontinued, the other can be used as an alternative. Despite the large number of compounds that has been tested in vitro, few have demonstrated an ability to induce a parasitological cure or alter the disease progression in animal models, and even fewer have advanced to clinical trials [19]. Nitro compounds remain some of the most promising options in combating diseases caused by parasites of the *Trypanosoma* genus. NFX has been also investigated as a potential treatment for pediatric tumors, such as neuroblastoma [20,21,22], and it was patented for treating cancer and inhibiting angiogenesis [23].

Among the faster strategies for improving the efficacy in Chagas disease and HAT treatment, drug combination strategies and the development of new formulations containing nitro-heterocyclic drugs are envisaged [24,25]. NFX has biopharmaceutical limitations, such as poor physicochemical stability, low solubility, erratic bioavailability, and fast metabolization, with consequent fast elimination (a half-life of 3 h) [26]. NFX’s solubility and dissolution rate limit its absorption [27,28]. The reformulation of NFX into a new dosage form may overcome these issues, but studies exploring this approach are scarce. Recently, our group has demonstrated that a self-emulsifying drug delivery system (SEDDS) provided advantages for dose adjustments to BZN with similar efficacy in an animal model [29]. SEDDSs are stable anhydrous formulations that improve drug absorption via the lymphatic pathway, reduce first-pass metabolism, and consequently improve bioavailability [30,31,32]. Thus, high plasma concentrations and reduced metabolization rates may be achieved. Additionally, BZN poly(ε-caprolactone) (PCL) implants improved the pharmacokinetic profile compared with that using classical BZN tablets in healthy and infected animals [33]. The main advantage of implant devices is the drug being released for an extended time—for example, for months—through the diffusion or degradation of its matrix by esterases in the body. PCL is a safe, stable, and low-cost biodegradable polymer [34,35,36] and is thus advantageous as a raw material for developing formulations for tropical neglected diseases that affect poor populations. Furthermore, implants in prolonged treatment are beneficial for maintaining doses and body exposure without the interruptions to treatment that occur frequently in patients suffering from Chagas disease. Therefore, these issues are the main challenges in NFX chemotherapy in Chagas disease, which could be circumvented by reformulating NFX.

This study aims to investigate the potential of implants and SEDDS to modify the efficacy of NFX. A lipid-based formulation for oral administration (the SEDDS) and a polymeric subcutaneous implant formulation for long-term release were developed, characterized, and tested in a murine infection model of *T. cruzi*. Both could be beneficial for the long treatment durations in this challenging antiparasitic chemotherapy, improving patient compliance.

## 2. Materials and Methods

### 2.1. Drugs and Materials

NFX was donated by the Drug for Neglected Diseases initiative (DND*i*). Cyclophosphamide (*N*,*N*-bis(2-chloroethyl)-1,3,2-oxazaphosphinan-2-amine-2-oxide (Genuxal) was purchased from Baxter (Baxter Oncology GmbH, Bielefeld, Germany, Germany). HPLC-grade acetonitrile, acetone, ethanol, and dimethyl sulfoxide (DMSO) were provided by Tedia^®^ (Rio de Janeiro, Brazil). Miglyol^®^810N (medium-chain triglycerides) was provided by Sasol GmbH (Sasol Germany GmbH, Brunsbüttel, Germany). Lecithin containing 70% phosphatidylcholine (Lipoid^®^S75) was provided by Lipoid GmbH (Frigenstrasse, Germany). Food-grade soy and sunflower oil was purchased from Liza^®^ (Cargill, Uberlandia, Brazil). Lipoid S75^®^ containing approximately 75% phosphatidylcholine was purchased from Lipoid GmbH (Ludwigshafen am Rhein, Germany). Tween^®^80, sodium phosphate monobasic, sodium hydroxide, hydrochloric acid, *N*,*N*-dimethylacetamide (DMA), and *N*-methyl pyrrolidone (NMP) were purchased from Vetec (Vetec Química Fina Ltda, Xerém, RJ, Brazil). Labrasol^®^ and Capryol 90^®^ were purchased from Gattefossé (Saint Priest, France), and Cremophor^®^EL was provided by BASF (Ludwigshafen am Rhein, Germany). Ultra-purified water was obtained using the Symplicity^®^185 system (Millipore, Bedford, MA, USA). Trypsin, fetal bovine serum (FBS), l-glutamine, penicillin/streptomycin, and Dulbecco’s Modified Eagle’s Medium (DMEM) as the cell culture medium were purchased from Sigma-Aldrich Co (St Louis, MO, USA). The poly-ε-caprolactone polymer (PCL; Mw 65,000 and Mn 42,500 g/mol, Ð 1.529 at 25 °C) was purchased from Sigma (Sigma-Aldrich Co., St. Louis, MO, USA).

### 2.2. The Chromatographic Conditions and Determination of Nifurtimox’s Solubility and Log P 

NFX quantification was performed using a Waters Alliance e2695 High-Performance Liquid Chromatograph (HPLC) with a Waters 2489 UV detector. The analysis employed a C18 Phenomenex^®^ Gemini column (150 × 4.6 mm, 5 μm) and a C18 Phenomenex^®^ pre-column (2 mm × 4.6 mm, 3 μm). The mobile phase consisted of an acetonitrile/water mixture (80:20 *v*/*v*), using isocratic elution at a flow rate of 0.80 mL/min, an injection volume of 25 μL, and a column temperature of 25°C. Detection was performed at 400 nm, the maximal absorption wavelength, which was chosen after a diode array detector (HPLC-DAD) scan from 210 to 500 nm. The NFX standard (purity > 98%) was obtained from Sigma-Aldrich™. The selectivity, specificity, linearity, precision, accuracy, detection limit, and quantification limit parameters were evaluated.

To improve the solubility of NFX in the analytical samples, many solvents were tested—for example, chloroform, dimethylacetamide, ethanol, hexane, methanol, *N*-methyl pyrrolidone, tetrahydrofuran, *n*-octanol, ethyl acetate, acetone, and benzyl alcohol. Solubility studies were also conducted in aqueous media, ultrapure water, and phosphate buffer saline (PBS). An excess of the drug (20 mg) was added to 1 mL of each solvent and incubated in a water bath at 37 °C for 48 h under magnetic agitation (SPLabor Multi-position Magnetic Stirrer with Heater). The mixtures were then centrifuged at 10,000 rpm for 20 min. The supernatant was carefully collected (100 μL) and filtered through 0.45 μm filters (Millipore). A 10 μL aliquot of the filtrate was diluted in 990 μL of acetonitrile for subsequent analysis using HPLC-UV/Vis.

The partition coefficient of NFX was determined using the moderate agitation method, validated for low-aqueous-solubility products [37]. *N*-octanol was used as the organic phase, and distilled water or phosphate-buffered saline (PBS) was used as the aqueous phase. The concentration of the drug in each phase was determined using HPLC-UV using the method described above. The experiment was performed in triplicate. The partition coefficient was expressed as the base 10 logarithm of the ratio between the drug concentration in the *n*-octanol and in the aqueous phases [log *P*].

### 2.3. Preparation and Characterization of the Nifurtimox Self-Emulsifying Delivery System (NFX-SEDDS)

The SEDDS was developed based on data on NFX’s solubility available in Supplementary Materials. The final composition was Miglyol 810N:Capryol 90:Lipoid S75:Labrasol:*N*-methyl pyrrolidone (NMP) (30:15:20:15:20) (% *v*/*v*). The SEDDS formulation was based on a BZN-loaded SEDDS previously developed, which was prepared under stirring and heating, as described by Mazzeti et al., 2020 [29]. To characterize the SEDDS, the following parameters were evaluated: the mean globule hydrodynamic diameter, the polydispersity index, and the zeta potential determined using Zetasizer PN3702 equipment (Malvern Instruments, Malvern, UK) as previously reported [29,38]. The NFX content in the formulation was evaluated using HPLC-UV/Vis according to the conditions validated above. Centrifugation tests were also performed to evaluate the stability of the emulsions formed after the dilution of the SEDDS in water. Furthermore, the effect of storage time on the physical stability of the SEDDS was also evaluated following a method described previously [29].

### 2.4. The Nifurtimox Release Kinetics In Vitro

The release kinetics of NFX from the NFX-SEDDS were evaluated using two different media, simulated gastric fluid (pH 1.2 ± 0.05) and simulated intestinal fluid (pH 7.5 ± 0.05), prepared according to the United States Pharmacopeia [39] and previously described in detail [29,40]. Pre-emulsified NFX-SEDDS formulations (1:10) were placed into cellulose dialysis membranes (6.4 mm, SpectraPor^®^, Spectrum Labs, East Longmeadow, MA, USA) that had been pre-hydrated in Milli-Q water for 24 h. The amount of NFX incorporated was adjusted to the sink conditions (approximately 10% of the maximum solubility in the respective media). The dialysis membranes were sealed using nylon threads and immersed in vessels containing either the simulated gastric or intestinal fluid. The experiments were conducted at 37 °C under constant agitation. Samples were collected in the bulk media at predetermined time intervals (0, 5, 15, 30, 60, 120, 180, 240, and 300 min) and quantified using HPLC-UV. The removed sample volume in the external media was replaced with fresh medium to maintain the conditions [41]. For comparative purposes, the same procedure was applied using coarse dispersions of NFX prepared in a 0.5% methylcellulose solution, which were also placed into the dialysis membranes under identical conditions. The experiments were performed in triplicate, as described by Zhang et al. [41].

The release and dissolution kinetics of NFX from the SEDDS were calculated based on experimental data using the mathematical models demonstrating the best fitting, determined by the coefficient of determination (R2). The first-order, Higuchi, and Weibull models were applied using DDSolver 1.0 Add-in software for Excel [42]. Experimental data on the in vitro release studies are reported as the means ± standard deviations.

### 2.5. In Vitro Cytotoxicity to Host Cells

The cytotoxicity of the NFX-SEDDS was determined in H9c2 cells (American Type Culture Collection, ATCC: CRL 1446), a cardiomyoblast lineage from rats. The cells were seeded at 1 × 10^3^ cells/well into 96-well plates with 100 μL of media and incubated for 24 h. Afterward, the cells were treated with free NFX (powder of the raw material) prepared from DMSO stock solutions and diluted in culture medium, ensuring a final DMSO concentration below 0.5% to prevent host cell toxicity. The additional treatments included the NFX-SEDDS or an equivalent amount of a blank-SEDDS being emulsified and diluted in the culture medium [29]. Eight successive serial dilutions (1:2) were made, starting with 1000 μM of the drug. A cell toxicity control was established using eight 1:2 serial dilutions of DMSO, starting at a concentration of 20% (*v*/*v*). The cell viability was measured after 72 h using the Resazurin colorimetric assay [29,43]. A reduction of more than 30% in the cell viability was considered cytotoxic [44].

### 2.6. The Anti-T. cruzi Activity of the Nifurtmox-SEDDS (Amastigotes)

The in vitro anti-*T. cruzi* activity assays were performed using H9c2 cells infected by trypomastigotes of the *T. cruzi* Y strain, which were obtained from the supernatant of the infected cells [29,45]. In 24-well culture plates covered with 13 mm cover slips, at 1 × 10^4^ cells/well, after 24 h, the cells were infected with *T. cruzi* trypomastigotes at a 20:1 ratio of the parasites to the host cells. After 24 h, the cells were treated with either free NFX (prepared from DMSO stock solutions and diluted in culture medium), the NFX-SEDDS, or an equivalent amount of a blank-SEDDS, both of which were emulsified and diluted in the culture medium [29]. The highest concentration was 10 μM of NFX for all groups, and subsequently, seven serial dilutions (1:2) were performed. After 72 h of incubation at 37 °C and 5% CO_2_, the cells were washed and fixed with methanol and stained with Giemsa. Anti-*T. cruzi* activity was calculated based on the inhibition of cell infection by amastigote forms, compared to that with the untreated control using a microscopic analysis, counting at least 200 cells per sample. All of the experiments were performed in duplicate, with at least two replicates. The IC_50_ values were calculated using CalcuSyn 2.1 software (Biosoft, Kington, UK).

### 2.7. Preparation and Characterization of the Polymeric Implants Containing Nifurtimox (NFX-PCL)

Initially, the NFX implants were produced using different concentrations of PCL (50 or 75) via the casting method [33]. Solid semicircular implants were molded in glass molds. The doses of NFX incorporated into the PCL polymer were based on the standardized oral dose for mice (50 mg/kg), assuming an average body weight of 25 g. Specifically, NFX (25, 37.5, or 50 mg) was weighed into the glass molds, followed by the addition of 100 µL of acetone and PCL. This amount incorporated 20-, 30-, and 40-fold the daily dose of NFX per mouse into the PCL implants. The mixture was stirred and heated at 65 °C in a water bath under 50 rpm agitation until complete dissolution. Afterward, the acetone was evaporated under heating at 65 °C, allowing the implant to solidify in a chemical hood. Residual solvents were removed under reduced pressure in a desiccator for 96 h. Blank implants (without NFX) were also prepared using the same procedure. The final implants were semicircular in shape and had a diameter of 10 mm [33].

The thermal behaviors of NFX, the polymeric implants containing NFX (NFX-PCL), and the physical mixtures of PCL and NFX were determined using Differential Scanning Calorimetry (DSC) and thermo-optical analysis (TOA). The DSC analyses were performed using a Mettler Toledo DSC 822e instrument, using indium and zinc standards. Approximately 2 mg was prepared in sealed aluminum pans. The samples were analyzed in the 25–200 °C temperature range, with a heating rate of 10°C/min, under a nitrogen atmosphere, and at a flow rate of 50 mL/min. The thermo-optical analysis was performed using a Mettler Toledo FP900, with an FP90 processor and an FP82 hot stage. Images were captured using a Leica DM4000B optical microscope with a 10× objective (C Plan), providing a total magnification of 100×. For the NFX analysis, the temperature range was set between 175 and 195 °C, with a heating rate of 2 °C/min. For the physical mixtures of PCL and NTX, the analyses were conducted in two temperature ranges: the first between 55 and 75 °C and the second between 155 and 185 °C.

All of the X-ray diffraction (XRD) analyses were conducted using a Rigaku Miniflex 300 system. The diffractograms were collected under the following settings—an angle range of 2° to 45° 2θ, a voltage of 30 kV, a current of 10 mA, 0.02° increments, and a scan rate of 5°/min—using a high-speed D-Tex Ultra detector.

The scanning electron microscopy (SEM) analyses were performed on a JEOL JCM-6000 system. Samples were prepared on carbon double-sided tape and fixed onto aluminum stubs. The pure NFX samples were gold-coated using a Denton Vacuum Desk V system, whereas the implant samples were not coated. Images were captured under varying fields and conditions.

### 2.8. Anti-T. cruzi Efficacy Studies in Mice

Female Swiss mice (18–24 g) were housed at the animal facility (CCA) of the Federal University of Ouro Preto, Brazil, under a controlled temperature (22 ± 2 °C) and 12-h light/dark cycles. The animals had free access to water and food. All of the procedures complied with the Ethical Principles of Animal Experimentation (COBEA) and were approved by the UFOP Ethics Committee under protocols 2014/56 and 2015/54.

**Figure 2:**
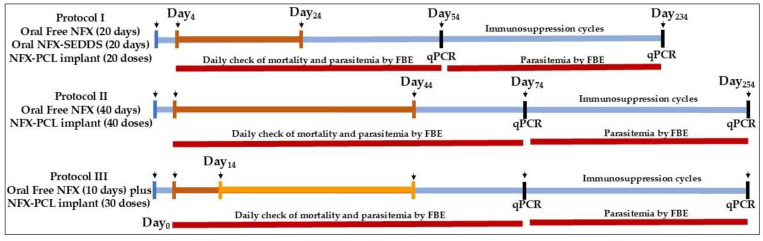
Experimental design of three treatment protocols: I (20 days), II (40 days), and III (mixed: oral plus implant). Non-infected and infected control groups are shown alongside groups receiving the different treatment regimens, including the oral administration of free nifurtimox (NFX), the NFX-SEDDS formulations, and the NFX-PCL implants. The treatment durations and evaluation time points for the mortality, parasitemia, and qPCR analyses are indicated. Blue lines represent the total duration of the experiment, brown and yellow lines indicate the NFX treatment duration, and red lines the time duration of parasitemia monitoring. FBE: fresh blood examination. qPCR: Polymerase Chain Reaction.

To determine the efficacy of the new formulations in an experimental murine model of infection, we inoculated the female mice via the intraperitoneal route with 5000 trypomastigotes of the Y strain of *T. cruzi*. This strain exhibits partial sensitivity to BNZ and NFX, achieving a 47% cure rate in mice treated orally from the 4th day post-infection for 20 consecutive days with 100 mg/kg of either BZN or NFX [46]. The treatments, via gavage or subcutaneous surgery for the implants, started on the 4th day of infection. The surgery was performed in anesthetized animals as per Mazzeti et al. [33]. After the implantation surgery, the animals were maintained in individual cages with access to food and water ad libitum. Subsequently, the animals were monitored for any signs of infection at the operative site or upon discomfort or distress. Any mice presenting with such signs were immediately euthanized. Complete healing at the site of surgery was observed in all animals. We also treated a control group of mice via the oral route with 50 mg/kg/day of NFX suspended in 0.5% (*w*/*v*) methylcellulose and administered via gavage. The experimental groups are shown in the schematic experimental design in Figure 2.

The animals were examined at least once a day to observe mortality and changes in clinical signs: appearance (twisting, piloerection, dirty eyes), pain (torsion, spasm), and behavior changes (withdrawal, vocalization, scratching, reluctance to move, irritability, anorexia, abnormal posture, ataxia).

The efficacy of the treatment was estimated by detecting parasites through fresh blood examination (FBE), using qPCR, and detecting mortality before and after the immunosuppression cycles, determined following the protocol previously described [47]. Briefly, mortality was checked daily until 30 days after the treatment. Fresh blood examination was performed daily to estimate the parasitemia during and up to 30 days after the end of the treatment, according to the Brener method [48]. Animals with negative results in the fresh blood examination were immunosuppressed using 50 mg/kg/day of cyclophosphamide (Baxter Oncology, Bielefeld, Germany) via the intraperitoneal route according to 3 cycles of 4 consecutive daily doses, with an interval of 3 days between each cycle. Parasitemia was checked daily during and up to 10 days after the end of the immunosuppression cycles (Figure 2).

The blood qPCR analyses were performed 30 and 180 days after the end of the treatment in samples from mice with negative fresh blood examination results. The Promega Wizard genomic DNA purification kit (United States) was used to extract the genomic DNA from 200 μL blood samples, according to the manufacturer’s instructions. The PCR analyses were performed using the primers TCZ-F (5=-GCTCTTGCCCACAMGGGTGC-3=, where M indicates A or C) and TCZ-R (5=-CCAAGCAGCGGATAGTTCAGG-3=), as described by [49]. The presence of *T. cruzi* in the blood samples was evaluated by amplifying a 195 bp tandem repeat in the genomic DNA [49]. The murine TNF-α gene sequence was amplified separately using the primers TNF-5241 (5=-TCCCTCTCATCAGTTCTATGGCCCA-3=) and TNF-5411 (5=-CAGCAAGCATCTATGCACTTAGACCCC-3=) [49]. For the reactions, 2 µL of template DNA (25 ng/µL), 10 µM of the primers, and 5 µL of Sybr-Green PCR Master Mix were used at a total volume of 10 µL. The amplifications were performed on a 7500 Fast Real-Time PCR System with 40 cycles at 94 °C for 15 s, followed by fluorescence data collection at 64.3 °C for 1 min. A melting curve analysis was conducted from 60 °C to 95 °C at 0.3 °C/s [47]. All of the samples were analyzed in duplicate, and negative samples and reagent controls were processed in parallel in each assay. Animals that showed negative results in all of the tests were considered cured.

### 2.9. Biochemical Toxicity Analysis

The toxicity of the treatments was evaluated through the hepatic enzyme dosages in the mouse serum collected on the last day of the treatment. Aspartate aminotransferase (AST) and alanine aminotransferase (ALT) were determined using a colorimetric assay using an autoanalyzer (Wiener Lab model CM200—kinetic analysis) and a commercial Bioclin^®^ kit according to the manufacturer’s instructions [29].

### 2.10. Statistical Analysis

Statistical treatment of the data was carried out using Graph Pad Prisma 5.01 statistical software (GraphPad Software Inc., San Diego, CA, USA). The results were expressed as the mean  ±  standard deviation. Parametric data were analyzed using Student’s *t*-test and nonparametric data using the Mann–Whitney test for the toxicity analysis. Statistical significance was established with 95% confidence intervals and *p* < 0.05.

## 3. Results

### 3.1. Nifurtimox Characterization

Determination of the apparent solubility of NFX in the different solvents was performed using HPLC/UV at 400 nm, with a retention time of approximately 2.3 min, using an acetonitrile/water (80:20) mobile phase. The parameters of the selectivity, specificity, linearity, precision, accuracy, limit of detection, and quantification were in accordance with the Guide of the International Conference on Harmonization [50]. The solubility of NFX in the different solvents is shown in Table 1. This test indicated that NFX is insoluble in water, with *N*-methyl pyrrolidone (NMP) being the most suitable solvent (90.85 mg/mL).

The partition coefficient provides information on molecules’ lipophilicity, which is closely related to their ability to passively cross biological membranes. The log *P* values for NFX in *n*-octanol/water were 0.87 ± 0.05 and 0.96 ± 0.07 in n-octanol/PBS. An in silico analysis using ALOGPS and Swiss ADME estimated log *P* values for NFX of 0.25 and 0.54, respectively. Log *P* values below 1.72 indicate low membrane permeability [52], suggesting that NFX likely exhibits a limited ability to permeate membranes. Considering the experimental low apparent solubility and log *P* results, following the Biopharmaceutics Classification System (BCS), NFX can be classified as class IV, i.e., a low-solubility and low-permeability drug.

### 3.2. Preparation and Characterization of the NFX-SEDDS

The SEDDS formulations self-emulsified immediately after their dilution in water. However, the droplets exhibited a broad size dispersion, as shown in Table 2. NFX incorporation into the SEDDS significantly increased the droplet size and polydispersity (*p* < 0.05) of the formulation. No significant effect of NFX on the zeta potential was evidenced. This result suggests NFX’s incorporation into the oily core of the emulsions, shielded by non-ionic surfactants like Labrasol.

When the blank-SEDDS formulations were emulsified in Milli-Q water (at a 1:10 ratio), nanoemulsions were formed. After centrifugation, no signs of creaming or phase separation were observed, indicating the stability of the nanoemulsions after their dispersion in water [29,53]. The physicochemical characteristics of the formulations were also evaluated monthly during their storage for six months. No NFX precipitate was detected in the anhydrous mixture during the evaluated period, and after emulsification, the original physical appearance of the formulations was maintained.

The physicochemical stability of the anhydrous SEDDS formulations enabled in vitro drug release studies comparing the NFX-SEDDS with a coarse suspension of NFX in 0.5% methylcellulose water (free NFX). NFX’s apparent solubility was first assessed in simulated gastric fluid and simulated intestinal fluid, measuring 0.094 ± 0.01 mg/mL and 0.089 ± 0.01 mg/mL, respectively.

**Figure 3:**
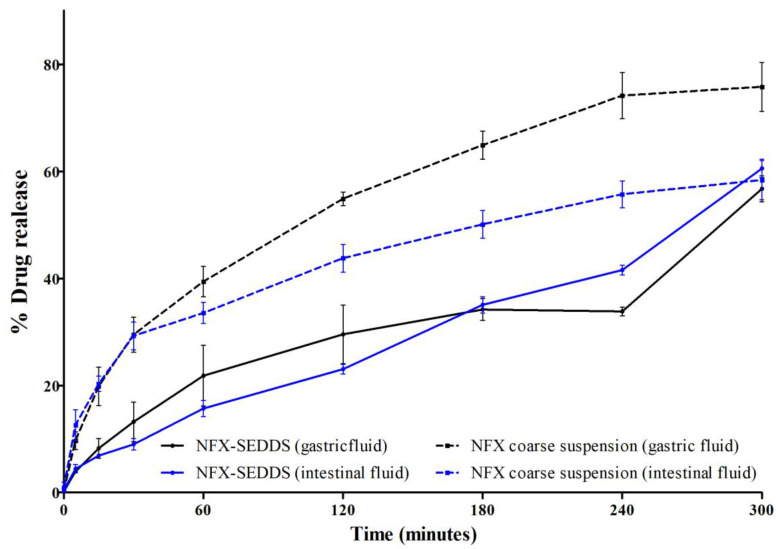
Comparative release profiles of nifurtimox from the SEDDS (solid lines) and the coarse suspension (dashed lines) in simulated gastric fluid (black) and intestinal fluid (blue) at 37 °C. The experiment was conducted under sink conditions using a direct dialysis method, and each time point represents the mean values and standard deviations (n = 3).

The dissolution profile, analyzed under sink conditions and quantified via HPLC-UV/Vis, revealed a faster drug release from the coarse suspension of NFX than that from the NFX-SEDDS in both gastric and intestinal fluids at up to 300 min. Previous reports show the complete dissolution of NFX from tablets in less than 120 min [28]. The NFX-SEDDS showed consistent NFX release regardless of the pH, highlighting its potential for controlled release. The NFX release from coarse suspension was slower in intestinal media compared to gastric media (Figure 3).

In the simulated gastric fluid, 50% of the NFX was released from the SEDDS after 5 h. In contrast, in the same period, almost 80% of the NFX was released from the coarse suspension, indicating that the SEDDS retained the drug inside the droplets for longer times in the gastric medium. Faster NFX release in the intestinal medium is observed for the coarse suspension compared with that for the SEDDS (Figure 3). Thus, the SEDDS formulations slow down the NFX fraction released in alkaline conditions, with more potential to activate lymphatic transport and circumvent the first-pass metabolism in the liver. None of the formulations achieved 100% release of the NFX during the evaluated period, confirming its classification as a BCS class IV drug.

The NFX release experimental data were mathematically modelized according to the first-order, Higuchi, and Weibull models because they provided the best fit for all of the formulations tested in vitro. The values of the release constants (K_1_, K_h_ and α, β) are shown, and the goodness of fit for each model was evaluated using statistical criteria, including the coefficient of determination (R2) and the Model Selection Criterion (MSC). The data were acquired for the NFX-SEDDS and the coarse suspension of NFX in order to compare the dissolution in the simulated gastric and intestinal fluid.

No statistical difference was observed among the SEDDS formulations at both pHs (SGF and SIF) in the first 120 min, in opposition to what occurred for the NFX suspensions. Thus, the incorporation of NFX into lipid droplets reduces the effects of pH on the release kinetics. The release constants in Table 3 show that the SEDDS reduces the NFX release rate compared with that with the coarse suspensions. The first-order and Higuchi models describes better the experimental data for SEDDS. Weibull mathematical model was better to describe the release data of the NFX suspensions. A significant difference (*p* < 0.05) was observed in the time required to release 75% of the NFX between the SEDDS and the suspensions. In the simulated intestinal fluid, the SEDDS excipients influenced the Weibull parameter β, indicating that the release curve had a sigmoidal profile (β > 1).

### 3.3. In Vitro Cytotoxicity and Anti-T. cruzi Activity of the NFX-SEDDS

After characterizing the physicochemical properties, stability, and drug release profiles of the SEDDS formulations, cytotoxicity tests were conducted to assess their effects on mammalian cells (host cells). From the dose–response curves, the 50% cytotoxicity concentrations (CC_50_) for the NFX and NFX-SEDDS were determined to exceed 30 µM for the H9c2 cells. Only non-toxic concentrations were used in the anti-*T. cruzi* activity experiments. The dose–response curves, evaluated in the H9c2 cells infected with the Y strain, of the free NFX and the NFX-SEDDS were similar, indicating that the formulation did not alter the drug’s activity. The blank-SEDDS showed no inhibitory effect on infection. The IC_50_ and IC_90_ values for the free NFX were 0.72 ± 0.15 µM and 6.88 ± 4.77 µM, respectively, comparable to those of the NFX-SEDDS, which were 0.71 ± 0.12 µM and 4.37 ± 1.44 µM (Table 4). The selectivity indices (SIs), calculated as the CC_50_/IC_50_ ratio, for the free NFX and the NFX-SEDDS were both greater than 45.

### 3.4. Characterization of the Polymeric Implants Containing Nifurtimox (NFX-PCL)

**Figure 4:**
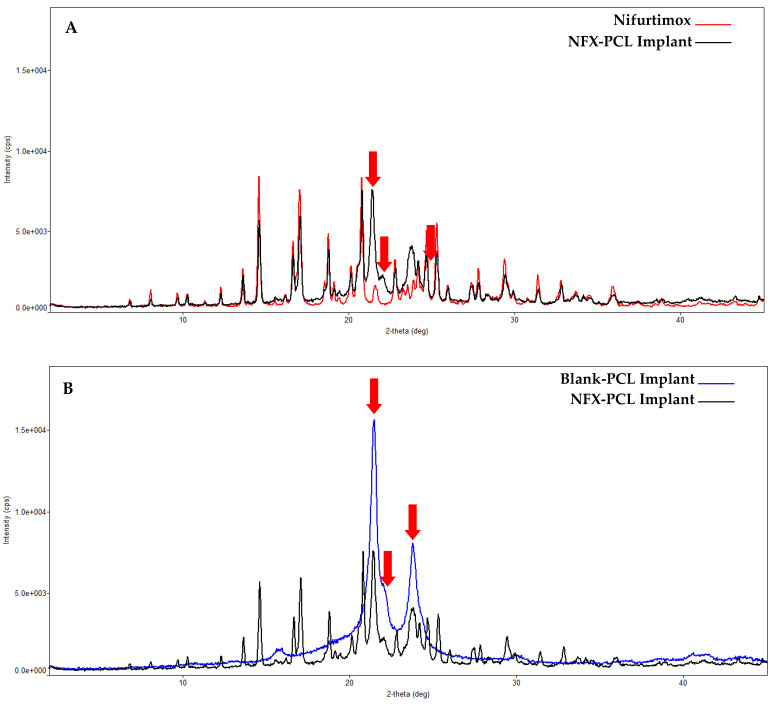
X-ray diffractogram analysis. (**A**) Overlay of X-ray diffractograms: NFX-PCL (in black) and nifurtimox (in red). (**B**) Overlay of X-ray diffractograms: NFX-PCL implant (in black) and blank-PCL implant (without the drug) (in blue). The red arrows show the peaks that cannot be attributed to the pure drug (in (**A**)) but can be attributed to the diffraction profile of the implant without NFX (in (**B**)).

NFX is a yellow to orange-yellow, odorless, crystalline powder [27]. The crystallographic data obtained in this study (Figure 4B) confirm the crystalline nature of the NFX, demonstrating that the sample used consisted of a single crystal form, closely resembling the theoretical profile stored in the Cambridge Structural Database under the code “JAHPEN” (qui^2^ = 1.8014) as previously reported [54]. The main (most intense) diffraction peaks were observed at angular positions of 14.575°, 17.041°, and 20.757° 2θ.

**Figure 5:**
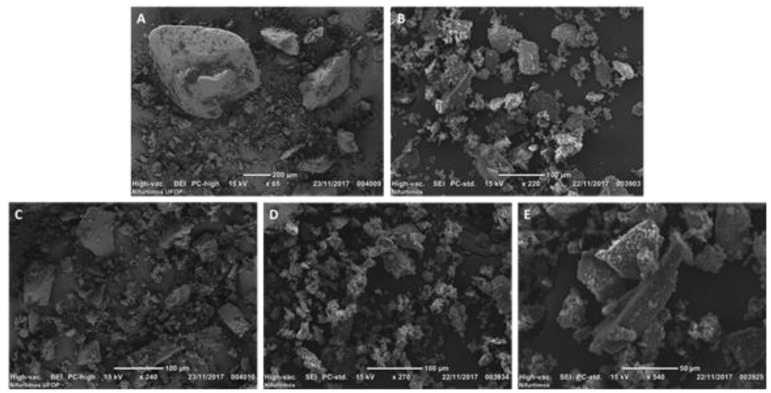
Scanning electron micrographs of nifurtimox: (**A**) magnification of 65×; (**B**) magnification of 220×; (**C**) magnification of 240×; (**D**) magnification of 270×; and (**E**) magnification of 540×.

Scanning electron microscopy (SEM) confirmed the crystalline nature of the sample, revealing a heterogeneous morphology that included both block- and plate-like particles of varying sizes, indicating that the sample comprised particles with a similarly diverse size distribution (Figure 5).

**Figure 6:**
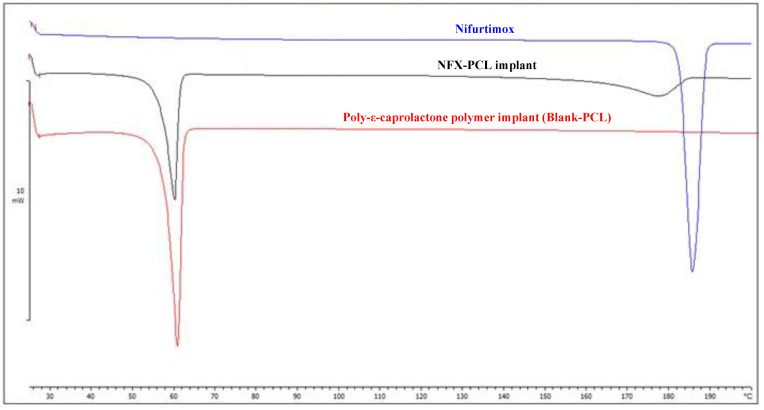
Differential Scanning Calorimetry (DSC) curve for pure nifurtimox (blue), the poly-ε-caprolactone polymer implant without the drug (blank-PCL) (red), and the nifurtimox–PCL implant (NFX-PCL).

**Figure 7:**
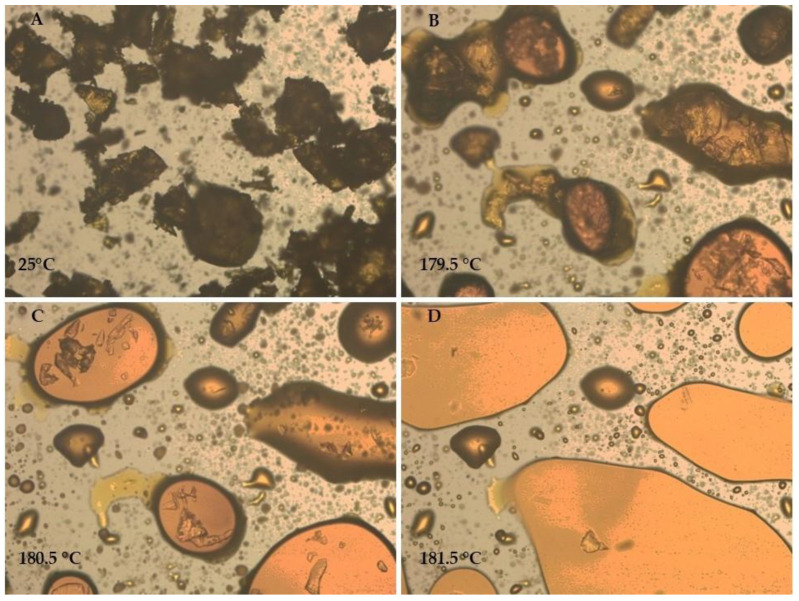
Thermo-optical analysis of pure nifurtimox crystal. (**A**) 25 °C, (**B**) 179.5 °C, (**C**) 180.5 °C, and (**D**) 181.5 °C. The images were captured using a Leica C Plan 10× objective lens, providing a total magnification of 100×.

The thermal profile of NFX was evaluated using Differential Scanning Calorimetry (DSC) (Figure 6) and thermo-optical analysis (TOA) (Figure 7). DSC revealed a single endothermic event corresponding to the NFX melting, occurring between 182.5 °C and 188.8 °C with a peak at 185.3 °C and an enthalpy of −109.04 J/g (Figure 6). The observation of a single melting event corroborates the X-ray diffraction (XRD) analysis, demonstrating that the sample contains a single crystalline phase. The TOA results showed slight variation, with fusion starting at 179.5 °C (Figure 7). At 181.5 °C, it was already possible to observe the total fusion of the particles in the sample. These differences can be attributed to the distinct analytical conditions of each method. The melting range, 180–182 °C, was consistent with data from the literature [27,55].

Figure 4A illustrates an overlay of an X-ray diffractogram of the NFX-PCL implant with that of pure NFX, highlighting the main diffraction peaks at 14.575°, 17.041°, and 20.757° 2θ. Figure 4B further shows an overlay with a diffractogram of the blank-PCL implant (without the drug). This analysis reveals that all of the diffraction peaks detected in the NFX-PCL implant corresponded directly to those observed in the separate analyses. This indicates that following the preparation of the implant, both the drug and the polymer maintained their crystalline and semicrystalline states, respectively, without chemical reactions or strong physical interactions. Therefore, under the analytical conditions employed, these results suggest that the method of implant preparation did not induce a polymorphic transition in the NFX, nor did it lead to its transformation into an amorphous solid state.

**Figure 8:**
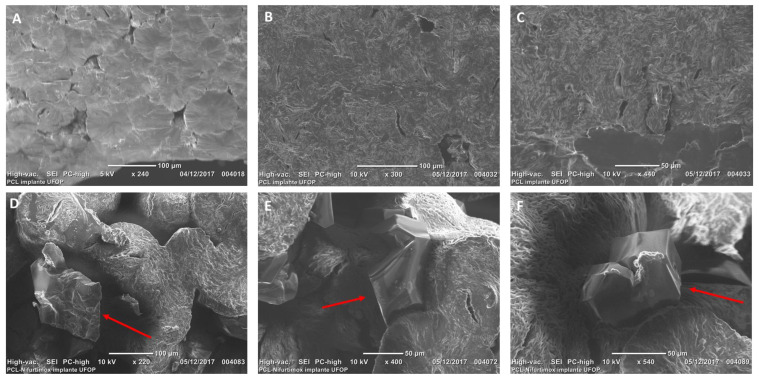
Scanning electron micrographs of the PCL implant without the drug (blank-PCL): (**A**) magnification at 240×; (**B**) magnification at 300×; and (**C**) magnification at 440×. Scanning electron micrographs of the NFX-PCL implant with red arrows indicates the NFX crystals: (**D**) magnification at 220×; (**E**) magnification at 400×; and (**F**) magnification at 540×.

The SEM analyses further corroborate the results obtained using XRD, demonstrating that after the implant preparation, the drug remained in its crystalline state, dispersed within the polymer matrix. Figure 8A–C show scanning electron micrographs of the pure PCL implant (without the drug), where its porosity is visible, without the presence of defined crystalline structures. In contrast, Figure 8D–F display micrographs of the NFX-PCL implant, which also exhibits a porous structure. However, in these images (indicated by the red arrows), defined particles with a distinct morphology, absent from the pure PCL implant, can be observed and are likely attributed to intact NFX crystals.

Although the XRD and SEM analyses show that both the PCL and NFX retain their structures when they are combined in implant form, the DSC analyses indicate a certain degree of interaction between these components. Figure 6 presents the DSC curve of the NFX-PCL implant overlaid with the DSC curves obtained for the pure NFX and the blank-PCL implant. The DSC curve of the NFX-PCL implant shows two endothermic events. In the first event, corresponding to the melting of PCL, no variation in the melting range, peak temperature, or expected enthalpy was observed compared to the melting event in the DSC curve for the blank-PCL implant. The second event on the NFX-PCL’s DSC curve can be attributed to the melting of the NFX within the implant. Observing this melting event confirms the XRD results, indicating that the drug remains in its crystalline state after implant preparation. However, this event is shifted and broadened, occurring between 164.9 °C and 184.3 °C with a peak at 177.5 °C. Furthermore, the enthalpy of this event was lower than expected: representing 40% of the formulation, the expected enthalpy of the NFX in the implant would be approximately −43 J/g. However, the observed value was −25.4 J/g, suggesting an interaction between the drug and the polymeric matrix.

**Figure 9:**
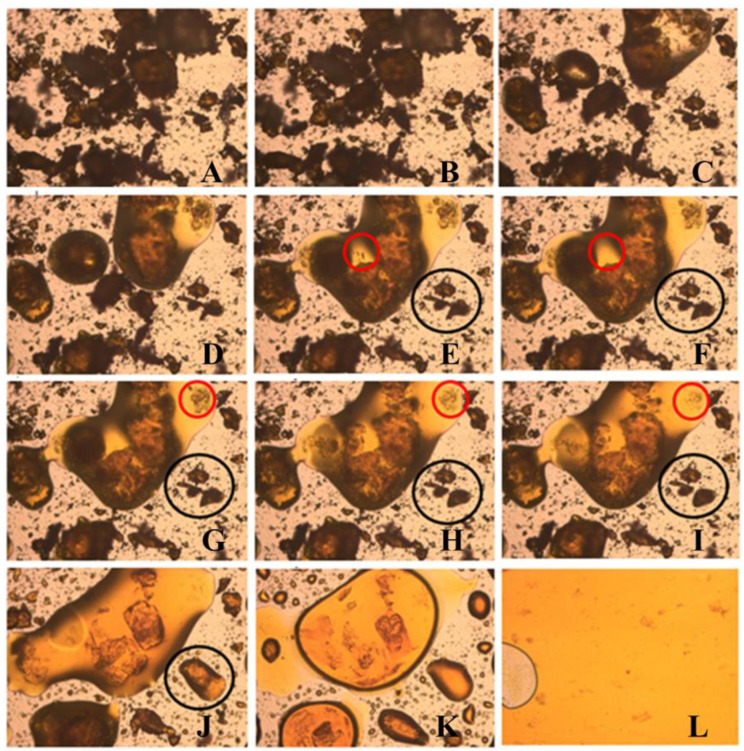
Thermo-optical analysis of the physical mixture of PCL and nifurtimox. The red circles highlight nifurtimox particles that solubilized into the polymer matrix upon heating, indicating a potential physical interaction, leading to solubilization. The black circles indicate nifurtimox particles that, without direct contact with the polymer, melted at the expected temperature for the pure drug, suggesting a lack of interaction in these regions. The temperatures correspond to specific thermal stages: (**A**) 25 °C, (**B**) 60 °C, (**C**) 66 °C, (**D**) 155 °C, (**E**) 160 °C, (**F**) 165 °C, (**G**) 168 °C, (**H**) 172.5 °C, (**I**) 175 °C, (**J**) 179.5 °C, (**K**) 180.5 °C, and (**L**) 181.5 °C.

To determine whether the changes in enthalpy observed in the DSC analysis were due to interactions between the substances, a TOA of the pure NFX and the NFX plus PCL mixture was conducted. In Figure 9, the TOA of the NFX plus PCL mixture shows the polymer melting between 60 °C and 66 °C. At 155 °C, the NFX particles began to be redistributed in the melted polymer. Small particles dissolved first, indicating partial solubility of the drug in the molten polymer matrix, suggesting a physical, rather than a chemical, interaction. The larger drug particles began to melt at 179.5 °C and fully fused at 181.5 °C, similar to the pure NFX. The TOA results align with the DSC findings, where the fusion range of the NFX remained unchanged but the enthalpy was reduced, possibly due to partial solubilization of the drug into the PCL polymer.

### 3.5. Anti-T. cruzi Efficacy of the SEDDS and the PCL Implants Containing Nifurtimox

To evaluate the treatment efficacy, Swiss mice were intraperitoneally inoculated with the partially benznidazole-sensitive Y strain of *T. cruzi*, and the treatments began on the fourth day post-infection, administered orally (via gavage) or through subcutaneous implantation of the drug-loaded devices. Post-surgical monitoring showed no signs of infection, discomfort, or distress, and all surgical sites healed completely. This approach allowed for a controlled evaluation of treatment effectiveness. All of the treatments with NFX successfully prevented mortality, which is otherwise observed in 100% of untreated infected animals.

The animals treated with either the free NFX or NFX formulations exhibited no changes in their clinical signs, appearance, or behavior or indications of pain. The infected mice treated with the blank-SEDDS or the blank-PCL implants showed outcomes similar to those for the untreated group, with no suppression of parasitemia or prevention of mortality. All of the treatments with NFX successfully prevented mortality, which is otherwise observed in 100% of untreated infected animals.

The SEDDS formulations demonstrated a similar in vivo efficacy to that of free NFX (*p* > 0.05) in reducing parasitemia in the *T. cruzi*-infected mice. Immunosuppression and qPCR confirmed cure rates of 28.6% for the NFX-SEDDS, highlighting its therapeutic potential compared to that of free NFX at the same dose (Table 5).

Initially, implants containing 50 or 75 mg of PCL and 25 mg of NFX (as the oral treatment with 50 mg/kg/day for 20 days) were evaluated in terms of their drug release (Table 5). Both formulations effectively suppressed parasitemia, but the 75 mg PCL implant (the NFX-PCL implant for 20 days) provided more sustained suppression, delaying parasitemia reactivation. The mice that received the 75 mg PCL implants (the NFX-PCL implant for 20 days) showed reactivation at 25 ± 11.45 days, while the 50 mg PCL group experienced reactivation at 10.67 ± 8.85 days. Oral treatment with 50 mg/kg of free NFX for 20 days resulted in parasitemia reactivation after 24.67 ± 4.14 days. Increasing the NFX dosage in the implant to 50 mg of NFX + 75 mg of PCL (the NFX-PCL implant for 40 days) reduced the parasitemia reactivation to 20%, with no reactivation in the mice treated orally for 40 days. Administering oral NFX (50 mg/kg/day) for 10 days, followed by using a 37.5 mg NFX + 75 mg PCL implant (NFX-PCL implant for 30 days), resulted in parasitemia suppression in 2 ± 1 days.

The cure rates varied by the administration route and duration. Oral NFX for 40 days cured 42.9% of the mice, similarly to the 40% cure rate for the NFX-PCL implant for 40 days. Shorter treatment durations resulted in lower cure rates for all of the NFX treatments and dosage forms (Table 5). The efficacy in this mice model is time- and dose-dependent, and the higher the duration of treatment, the higher the efficacy.

**Figure 10:**
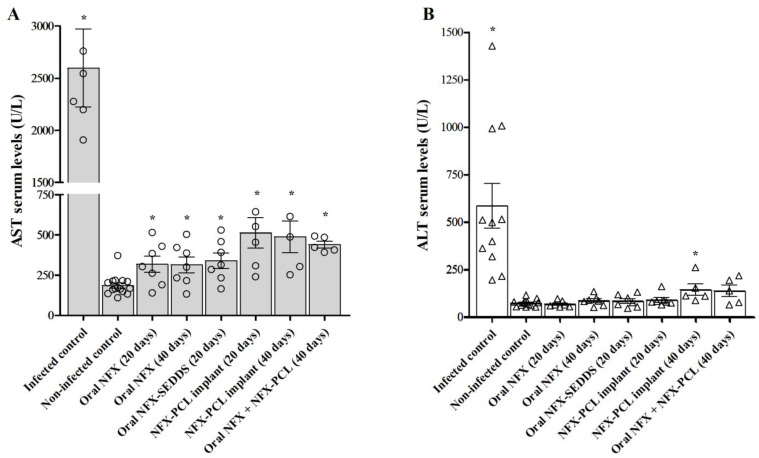
Liver enzymes levels of treated mice. (**A**) Aspartate aminotransferase (AST) and (**B**) alanine aminotransferase (ALT) serum levels of non-infected and mice infected with the *Trypanosoma cruzi* Y strain treated for 20 or 40 days with nifurtimox (NFX), the NFX-SEDDS, or NFX-PCL implants. Samples were collected on the last day of treatment. The levels of AST and ALT in the infected and non-treated control groups were measured on the 15th day of infection. * Different compared to the non-infected control, at *p* < 0.01.

Liver enzyme levels, specifically AST and ALT, were measured in the serum of the animals on the final day of treatment. The levels of AST and ALT in the infected untreated control groups were assessed on the 15th day post-infection, prior to mortality caused by the Y strain of *T. cruzi*. Figure 10 presents the hepatic enzyme levels in the animals treated with different formulations of NFX. All treatments significantly reduced their AST and ALT levels compared to those in the untreated infected animals, indicating a decrease in tissue damage, which may be related to a reduction in the parasite load in the hepatic tissues.

**Figure 11:**
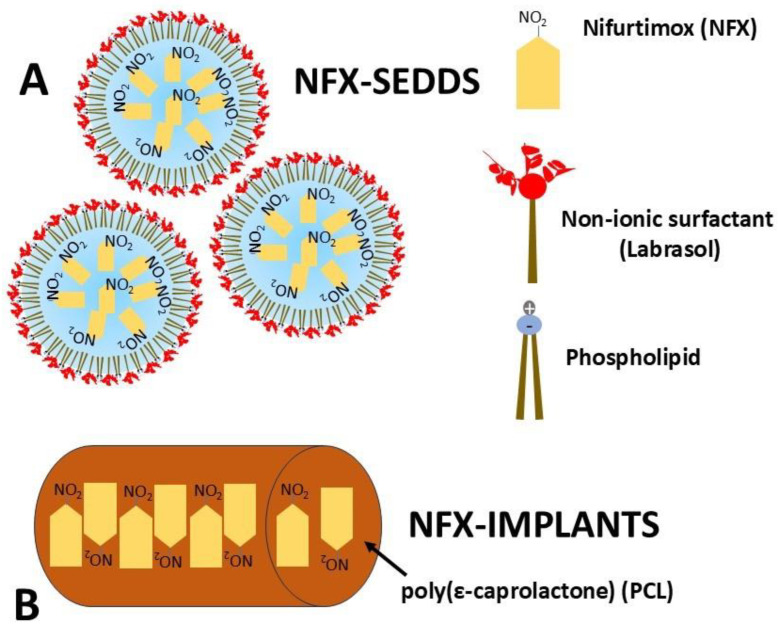
Schematic representation of the nifurtimox delivery systems. (**A**) Schematic representation of droplets of nanoemulsions formed after the self-emulsifying system (SEDDS)’s dispersion in water in the gastrointestinal tract; (**B**) subcutaneous implants with nifurtimox.

Figure 11 presents a schematic representation of the NFX delivery systems. Panel A illustrates the NFX-SEDDS, where NFX molecules were incorporated into micelles stabilized by phospholipids and the non-ionic surfactant Labrasol. This structure facilitates drug solubilization and potentially enhances bioavailability. Panel B shows the NFX-PCL implants, depicting the drug dispersed within the polymeric matrix. These innovative formulations aim to enhance NFX’s solubility, bioavailability, and sustained release for improved therapeutic efficacy.

## 4. Discussion

Although NFX is one of the only two drugs available for the treatment of Chagas disease and used for HAT treatment, few details about the physicochemical characterization of this active pharmaceutical ingredient (API) are available [27]. The results of this study provide new insights into the development of innovative formulations of NFX for the treatment of Chagas disease. This is particularly significant given the limitations of the current therapies, which include low efficacy in the chronic phase of infection and significant adverse effects that hinder patient adherence [18,56]. Two formulations, a lipid-based self-emulsifying drug delivery system (SEDDS) and poly(ε-caprolactone) (PCL) implants, were investigated for their potential to address these biopharmaceutical issues.

In the solubility tests, NFX demonstrated high solubility in NMP, a water-miscible, aprotic solvent, approved for use in the solubilization of drugs for oral and topical administration [57]. This solvent is useful for the solubilization of nitrofurans in assays in vitro and in vivo. Based on its water solubility and log *P* results, NFX can be classified as a BCS class IV drug, exhibiting low solubility and low permeability. These are critical and challenging physicochemical factors to consider in the development of new pharmaceutical formulations. In contrast, Kasim and co-authors estimated NFX’s cLog P and solubility in water (33 mg/mL) and categorized the substance into class III (high-solubility and low-permeability) [51]. Our result in the present work differs greatly. In addition, NFX has previously been categorized as class II (low-solubility, high-permeability) and class IV (low-solubility, low-permeability) [27]. The contradictions observed may reflect the source of the data, the crystal forms, and the experimental and quantification methods used.

The NFX sample used in this study consisted of a single crystalline phase [27,57]. NFX’s thermal characteristics observed in this study also correspond to those described by Moroni et al. (2023), who reported a melting peak at 181.8 °C in their DSC analysis and 178 °C in their thermo-optical analysis [27].

In vitro release tests are crucial for estimating the amount of the drug released when it is in contact with various physiological fluids via oral administration. The characterization of the SEDDS formulations revealed the improved dispersibility of NFX in the SEDDS after colloidal dispersion in water. The SEDDS maintains a consistent release rate across pH variations, a critical factor in overcoming the variable gastrointestinal environment, since the bioavailability of NFX is diet-dependent and erratic [26,28]. However, a slower release was observed, indicating that the SEDDS may retain NFX in the alkaline media inside nanodroplets. This may activate the lymphatic transport through the intestinal membrane, bypassing the first-pass metabolism [58,59]. If this absorption mechanism occurs, it may have effects on the elimination rate and eventually on efficacy. This point merits further investigation in future studies. The in vitro release studies helped correlate the percentage of the drug solubilized with its availability for absorption in vivo. However, the correlation between in vitro and in vivo assays is often weak due to the limitations of in vitro experiments, particularly in replicating the complexity of the gastrointestinal tract. In the case of SEDDS formulations, not only dispersion but also intestinal transit and lipid digestion by gastric and pancreatic lipases play a significant role in maintaining a drug in its solubilized form [60].

The cytotoxicity assays demonstrated that the NFX-SEDDS formulation was non-toxic at therapeutic concentrations, with its selectivity indices comparable to or better than those of free NFX. The anti-*T. cruzi* activity was preserved in the NFX-SEDDS, with the IC_50_ values remaining within the expected ranges. This highlights the potential of these formulations to maintain or even enhance this drug’s efficacy while reducing its systemic toxicity. These findings emphasize the importance of maintaining drug efficacy while minimizing host toxicity and consequently diminishing the general side effects for Chagas disease patients. Another advantage of the SEDDS formulation is the possibility of personalized dosing and administration in liquid form, which would facilitate treatment in pediatrics [28,29,61].

The characterization of the NFX-PCL implant indicates that NFX retains its crystalline structure within the implant, which is relevant from the perspective of its pharmacokinetic properties. Although the thermal analyses indicate that some degree of physical interaction occurs between the polymer and the drug—likely due to the partial solubilization of nifurtimox in the polymer matrix—it should be noted that this effect is observed under extreme temperature conditions. Nevertheless, these results suggest the presence of a physical, rather than a chemical, interaction between NFX and PCL. Possible chemical interactions, which could lead to the formation of potential drug degradation products, should still be evaluated in long-term stability studies. These minor physical interactions suggested some degree of solubilization, which might influence the NFX’s release kinetics.

In vivo studies further evidenced the clinical potential of these formulations. The NFX-SEDDS provided parasitemia suppression comparable to that of the administration of free NFX. The NFX-PCL implants demonstrated sustained release over extended periods (20 days), potentially improving the patient adherence and treatment outcomes, as has already been demonstrated using benznidazole implants [33]. Although the cure rates were similar between the oral and implant formulations in mice model, the implants’ convenience and ability to maintain therapeutic levels for extended durations represent a significant advantage, especially for chronic-phase patients. Chagas disease is predominantly treated during the chronic phase, a stage where the parasite’s replication slows and the symptoms often progress to severe cardiac or gastrointestinal complications. Treatment during this phase presents significant challenges, particularly related to patient adherence [62,63].

Regarding the toxicological profile of the formulations, the potential for toxicity to be induced by the formulation excipients, and the high NFX content incorporated into the implants, we evaluated the hepatic damage during treatment. The levels of the hepatic enzymes AST and ALT were measured in the serum of the animals on the last day of treatment. All treatments significantly reduced their AST and ALT levels in comparison to those in untreated infected animals. Furthermore, treatment with the various formulations, even over prolonged periods, did not interfere significantly with enzyme levels. This finding suggests treatment safety, potentially related to a reduction in the parasitic load in the liver [64]. In addition, a recent study showed the excellent biocompatibility and minimal cytotoxicity of PCL. PCL scaffolds implanted into male Wistar rats caused no signs of inflammation, tissue damage, or systemic toxicity [65]. These results underscore its potential for biomedical applications, including implantable drug delivery systems.

NFX faces significant limitations that necessitate the development of novel formulations. As a BCS class IV drug, its low solubility and permeability severely restrict its bioavailability and therapeutic efficacy [24]. Additionally, concerns related to its toxicity, narrow safety margin, and high interpatient variability in absorption further complicate its clinical application [24,61]. Previous strategies to enhance NFX delivery, including nanocarriers such as liposomes and polymeric nanoparticles, have shown potential in improving its solubility and protecting the drug from degradation under physiological conditions [24]. However, these approaches are hindered by challenges such as complex manufacturing processes, stability concerns, and difficulties in scaling up production. Clinical studies evaluating the biopharmaceutical characteristics of NFX tablets underscore the urgent need for strategies to enhance its dissolution and bioavailability [61]. Innovative formulations, including PCL implants and optimized SEDDSs, as investigated in this study, address these challenges by offering improved solubility, sustained release profiles, and better therapeutic outcomes, effectively overcoming the limitations of the conventional NFX delivery methods.

## 5. Conclusions

This study highlights the significant potential of NFX-SEDDSs and NFX-PCL implants as innovative drug delivery systems for Chagas disease, addressing the major limitations of the current treatments. The SEDDS formulations improved the drug’s solubility and dispersibility in gastrointestinal fluid and reduced its systemic toxicity. These lipid-based systems have the potential to improve lymphatic absorption and reduce the first-pass metabolism, a notable advantage in maintaining therapeutic concentrations. The characterization of the implants confirmed the retention of NFX in its crystalline state, with minor physical interactions, which could enhance release kinetics without compromising stability. The sustained-release property of the implants offers a promising alternative for long-term therapy, being particularly suitable for chronic-phase Chagas disease, where patient adherence is critical. The use of a biocompatible polymer, such as PCL, minimizes the local and systemic toxicity while maintaining an efficacy comparable to that of the standard oral regimens. This approach aligns with the growing interest in new formulation strategies for neglected tropical diseases.

Future research should expand the tests with these formulations into chronic infection models and evaluate their applicability in strains with different susceptibility profiles. Additionally, long-term stability studies and pharmacokinetic modeling are essential to fully characterize the clinical potential of these systems. Combining pharmaceutical innovation with practical treatment solutions offers a path to improving patient outcomes and addressing the global health burden of Chagas disease. These findings also open new avenues for adapting similar technologies to other nitro-heterocyclic drugs used in the management of trypanosomatid-based infections, further broadening their therapeutic impact.

## Figures and Tables

**Table 1:** Apparent solubility of nifurtimox in different solvents.

Solvent	Solubility (mg/mL)	Classification *
*N*-methyl pyrrolidone	90.85 ± 9.04	Soluble
Acetone	17.27 ± 0.28	Slightly soluble
Chloroform	14.03 ± 2.65	Slightly soluble
Acetonitrile	12.57 ± 3.48	Slightly soluble
Ethyl acetate	11.90 ± 0.87	Slightly soluble
Benzyl alcohol	10.97 ± 1.12	Slightly soluble
Tetrahydrofuran	5.08 ± 3.48	Slightly soluble
Methanol	1.17 ± 0.09	Poorly soluble
Dimethylacetamide	0.89 ± 0.39	Very little soluble
Ethanol	0.65 ± 0.05	Very little soluble
n-Octanol	0.11 ± 0.00	Very little soluble
Hexane	0.001 ± 0.00	Insoluble
Phosphate-buffered saline (PBS)	0.04 ± 0.00	Insoluble
Water	0.05 ± 0.00	Insoluble

* Classification according to the International Pharmacopoeia and [51].

**Table 2:** Physicochemical characterization and stability of SEDDS droplets after self-emulsification.

Solvent	Blank-SEDDS	NFX-SEDDS
NFX visual precipitation	NA	no ppt
Droplet size (nm)	282.7 ± 13.0	612.57 ± 8.53
Polydispersity index (PdI)	0.498	0.730
Zeta potential (mV)	−46.6 ± 1.81	−46.0 ± 3.15
Nifurtimox (mg/mL)	NA	13.30 ± 0.80 *

Anhydrous SEDDS formulations were emulsified in ultra-pure water at 37 °C at 1:1000 dilution and stirred for 20 s. NFX = nifurtimox; NA = not applicable, ppt: NFX precipitates. * Content of NFX was determined using the HPLC method.

**Table 3:** Mathematical modeling of the benznidazole dissolution/release from the SEDDS in PBS at a pH of 6.8 and 37 °C.

Formulation	First-Order *F* = 100.(1 − *e*^−*k*^_1_^t^)				Higuchi *F* = 100.*k_h_*.*t*^−0.5^				Weibull *F* = 100.(1 − *e*^tβ/α^)			
	*K*_1_ (min^−1^)	RSD %	*MSC*	*R* ^2^	*K_h_* (min^−1/2^)	RSD %	*MSC*	*R* ^2^	*α*	*β*	*MSC*	*R* ^2^
NFX coarse suspension (SGF)	0.00550 ± 0.00093	16.98	1.986	0.8720	4.7340 ± 0.2914	6.15	3.512	0.9760	22.2552	0.591	4.660	0.9957
NFX-SEDDS (SGF)	0.00246 ± 0.00021	8.47	1.986	0.8642	2.7073 ± 0.3042	11.23	2.069	0.8923	54.0146	0.611	1.557	0.8835
NFX coarse suspension (SIF)	0.00348 ± 0.00042	12.04	0.649	0.6547	3.7386 ± 0.3030	8.10	2.305	0.9317	12.7247	0.468	4.392	0.9934
NFX-SEDDS (SIF)	0.00267 ± 0.00012	4.68	3.052	0.9608	2.7613 ± 0.0613	2.22	2.122	0.9032	67.1088	30.976	1.698	0.9057

α is the scale parameter that defines the time scale of the process; β is the shape parameter that characterizes the curve; F is the fraction dissolved; R^2^ is the coefficient of determination; MSC: Model Selection Criterion. Values > 2 indicate the goodness of fit. SGF: simulated gastric fluid. SIF: simulated intestinal fluid.

**Table 4:** Cytotoxicity and anti-*Trypanosoma cruzi* activity of nifurtimox and the NFX-SEDDS.

	Cell Viability	Anti-*T. cruzi* Activity		Selective Index (SI)
	CC_50_ (µM)	IC_50_ (µM)	IC_90_ (µM)	
Nifurtimox	34.73 ± 10.38	0.72 ± 0.15	6.88 ± 4.77	48.2
Blank-SEDDS	39.21 ± 9.03	ND	ND	-
NFX-SEDDS	46.86 ± 9.38	0.71 ± 0.1	4.37 ± 1.44	66

CC_50_ = cytotoxic concentration for 50% of the H9c2 cells. IC_50_ concentration inhibits 50% of the *T. cruzi* amastigotes. SI = CC_50_/IC_50_. The NFX, NFX-SEDDS, and blank-SEDDS were tested in H9c2 cells to determine cytotoxic concentration for 96 h, and for 72 h in H9c2 cells infected with *Trypanosoma cruzi* Y strain to determine activity and inhibitory concentration.

**Table 5:** Effects of nifurtimox, the NFX-SEDDS, and the NFX-PCL implants in different treatment regimens on the course of the mice’s infection with the Y strain of *Trypanosoma cruzi* ^1^.

Dosage Form	Groups/Protocol	Treatment Duration (Days)	Parasitemia Clearance/(Days of Treatment)	Negative Results—FBE and PCR/n° Animals ^2^	Number of Surviving/Total Animal
	Non-infected control	-	-	6/6 (100%)	6/6 (100%)
	Infected control	-	0/7 ND	0/7 (0%)	0/7 (0%)
SEDDS	Infected treated—blank-SEDDS	40	0/7 ND	0/7 (0%)	0/7 (0%)
Implant	Infected treated—blank-PCL implants	40	0/7 ND	0/7 (0%)	0/7 (0%)
Free	Oral NFX 50 mg/kg/day	20	7/7 (1.43 ± 0.79)	1/7 (14.3%)	1/7 (14%)
Free	Oral NFX 50 mg/kg/day	40	7/7 (1.71 ± 0.49)	3/7 (42.9%)	7/7 (100%)
SEDDS	Oral NFX-SEDDS 50 mg/kg/day	20	7/7 (1.71 ± 0.75)	2/7 (28.6%)	7/7 (100%)
Implant	25 mg NFX + 50 mg PCL	20	6/6 (1.67 ± 0.51)	0/6 (0%)	6/6 (100%)
Implant	25 mg NFX + 75 mg PCL	20	7/7 (2.00 ± 1.00)	0/7 (0%)	7/7 (100%)
Implant	50 mg NFX + 75 mg PCL	40	5/5 (1.20 ± 0.45)	2/5 (40%)	5/5 (100%)
Free oral + Implant	Oral NFX 50 mg/kg/day, 10 days + (37.5 mg NFX + 75 mg PCL), 30 days	40	5/5 (2.00 ± 1.00)	1/5 (20%)	5/5 (100%)

^1^ Swiss mice (18 to 22 g) were inoculated with 5 × 10^3^ trypomastigotes from the *Trypanosoma cruzi* Y strain. The treatment started 4 days after infection and continued for 20 or 40 days. ^2^ FBE (fresh blood examination) before and after immunosuppression using cyclophosphamide. PCR (Polymerase Chain Reaction) assays were performed 30 and 180 days after the treatment. NFX: nifurtimox. PCL: poly-Ɛ-caprolactone.

## Data Availability

The original contributions presented in this study are included in the article/Supplementary Material. Further inquiries can be directed to the corresponding authors.

## Supplementary Materials

The following supporting information can be downloaded at: https://www.mdpi.com/article/10.3390/tropicalmed10020050/s1, Table S1: Solubility of nifurtimox in different SEDDS excipients.
