# Supplementary material for: Development and Characterization of Innovative Nifurtimox Formulations as Therapeutic Alternative for Chagas Disease
Source: Trop Med Infect Dis. 2025 Feb 7;10(2):50. doi: 10.3390/tropicalmed10020050 (PMC11860281; doi:10.3390/tropicalmed10020050)
Supplement: Supplementary file 1 [file tropicalmed-10-00050-s001.zip › tropicalmed-3422161-supplementary.pdf]

Table S1 - Solubility of nifurtimox in different SEDDS excipients

	Excipient	Nifurtimox solubility (mg/ml)	HLB[1]
**Oil**	Miglyol 810®	1.05 ± 0.10	-
	Soy oil	0.30 ± 0.03	-
	Sunflower oil	0.16 ± 0.01	-
**Surfactant**	Capryol-90®	1.90 ± 0.11	5
	Cremophor® EL	3.15 ± 0.08	12 -14
	Labrasol®	5.51 ± 0.30	14
	Tween 80®	2.75 ± 0.55	15
	Lipoid®S75	ND	-

1 HLB= Hydrophilic Lipophilic Balance, ND= Not determined
